# Effects of *Laminaria japonica* Polysaccharide Supplementation in Aged Corn-Based Diets on Growth Performance, Nutrient Digestibility, Rumen Fermentation, and Muscle Antioxidant Status of Hu Sheep

**DOI:** 10.3390/ani16142278

**Published:** 2026-07-22

**Authors:** Huiwen Zheng, Weitong Peng, Jiamei Song, Yuansheng Ma, Hangshu Xin, Yonggen Zhang, Guangning Zhang

**Affiliations:** College of Animal Science and Technology, Northeast Agricultural University, Harbin 150030, China; 15590949571@163.com (H.Z.); 13793546812@163.com (W.P.); songjiamei1120123@163.com (J.S.); yuanshengma@126.com (Y.M.); hangshu.xin@neau.edu.cn (H.X.); zhangyonggen@neau.edu.cn (Y.Z.)

**Keywords:** aged corn, *Laminaria japonica* polysaccharides, Hu sheep, feed utilization efficiency, nutrient digestibility, rumen fermentation, muscle antioxidant status

## Abstract

Long-term storage can impair the feeding value of corn. This study evaluated whether dietary *Laminaria japonica* polysaccharide (LJP) could alleviate the adverse effects of aged corn in Hu sheep. Twenty-one male Hu sheep (39.05 ± 3.55 kg) were assigned to a normal-corn diet (NC), an aged-corn diet (AC), or the AC diet supplemented with 0.5% LJP (AC+LJP; *n* = 7) for 70 d, including a 14-d adaptation period. Compared with NC, AC reduced average daily gain, gain-to-feed ratio, the apparent digestibility of crude protein, neutral detergent fiber and acid detergent fiber, ruminal total volatile fatty acids, acetate and butyrate, and muscle glutathione peroxidase activity. LJP supplementation restored average daily gain, gain-to-feed ratio, and crude protein and neutral detergent fiber digestibility to levels comparable with NC. It also increased ruminal ammonia nitrogen, microbial crude protein, total volatile fatty acids, acetate, butyrate, and isobutyrate relative to AC, while reducing muscle malondialdehyde concentration and increasing total antioxidant capacity. Dietary treatment had little effect on carcass traits, most meat-quality variables, or overall rumen bacterial diversity. In conclusion, supplementation with 0.5% LJP partially alleviated the decline in nutrient utilization and rumen fermentation associated with aged corn and improved muscle antioxidant status. These findings support the potential use of LJP as a functional feed additive to improve the utilization of aged-corn-based diets in sheep.

## Simple Summary

Corn is an important energy source in sheep diets, but long-term storage can reduce its feeding value. This study evaluated whether *Laminaria japonica* polysaccharide (LJP), a seaweed-derived feed additive, could improve the utilization of aged corn by Hu sheep. Feeding aged corn reduced growth rate, feed efficiency, nutrient digestibility, rumen fermentation, and muscle antioxidant status. Supplementing the aged-corn diet with LJP at 0.5% of basal-diet DM improved growth and feed conversion, enhanced protein and fiber digestion and rumen fermentation, and strengthened muscle anti-oxidant capacity, while carcass traits and most meat-quality characteristics remained unchanged. These findings indicate that LJP supplementation may help sheep producers use aged corn more efficiently, provided that the corn meets feed-safety standards. However, larger-scale and longer-term studies are needed before broad commercial application.

## 1. Introduction

Corn is one of the most important energy feed ingredients in ruminant production systems because it provides large amounts of starch and fermentable carbohydrates required for ruminal microbial growth and host energy metabolism [1,2]. With the increasing emphasis on food security and strategic grain reserves worldwide, the long-term storage of corn has become increasingly common in many countries [3]. Consequently, a considerable proportion of stored corn may eventually enter animal feeding systems after prolonged storage periods. However, storage duration is an important factor affecting grain quality. During long-term storage, corn may undergo a series of physicochemical changes, including starch degradation or retrogradation, lipid hydrolysis, lipid oxidation, changes in protein and starch properties, and losses of biologically active nutrients, resulting in a gradual decline in nutritional value and storage stability [4,5]. These changes may influence the feeding value of corn even when visible spoilage is absent and mycotoxin concentrations remain within regulatory limits [6]. Therefore, understanding the nutritional consequences of feeding aged corn has become increasingly important for the efficient utilization of grain reserves in animal production systems.

Previous studies have demonstrated that storage-related deterioration of corn can negatively affect animal performance and physiological status. In monogastric animals, aged corn has been associated with reduced growth performance, impaired nutrient utilization, altered intestinal function, and increased oxidative stress [3]. The proposed mechanisms include reduced nutrient availability, oxidation of lipids and proteins, and increased metabolic burden caused by storage-related deterioration. In ruminants, however, the biological consequences of feeding aged corn remain less clearly understood. Because ruminal microorganisms depend on fermentable carbohydrates to produce volatile fatty acids (VFAs) and microbial protein, changes in starch availability and feed quality may directly influence ruminal fermentation efficiency, nutrient utilization, and ultimately animal performance [2,7]. Moreover, the oxidative deterioration of feed ingredients may affect systemic redox balance and tissue antioxidant status [8]. These observations suggest that aged corn may influence animal productivity through both nutritional and oxidative pathways.

Although increasing attention has been paid to the utilization of aged corn in animal feeding, several important knowledge gaps remain. First, most previous studies have focused on monogastric animals, whereas information regarding the effects of aged corn on sheep is limited [9]. Second, existing studies have generally evaluated only a limited number of response variables, such as growth performance or nutrient digestibility, making it difficult to clarify the relationships among nutrient utilization, ruminal fermentation, rumen microbial composition, and host physiological responses. Recent studies and reviews emphasize that diet-induced changes in rumen microbial composition and fermentation products are closely associated with feed efficiency, fermentation patterns, and host metabolism in ruminants [2,10]. Third, practical nutritional strategies to alleviate the adverse effects of aged corn have received relatively little attention. Consequently, it remains unclear whether interventions targeting ruminal fermentation and oxidative balance can effectively improve the utilization of aged-corn-based diets.

*Laminaria japonica* polysaccharide (LJP), a bioactive polysaccharide extracted from brown seaweed, has attracted increasing interest as a functional feed additive because of its antioxidant, immunomodulatory, and prebiotic properties [11,12]. Previous studies have shown that seaweed-derived polysaccharides can modulate gastrointestinal microbial communities, enhance fermentation efficiency, improve antioxidant status, and promote animal health [13,14]. Mechanistically, LJP may act as a fermentable and prebiotic substrate that modulates rumen microbial activity, thereby promoting carbohydrate and fiber degradation, microbial protein synthesis, and volatile fatty acid production. In addition, its antioxidant properties may facilitate the scavenging of reactive oxygen species and enhance endogenous antioxidant defenses, thereby helping to maintain redox homeostasis. In animal production, LJP supplementation has been reported to improve growth performance, digestive enzyme activity, gut microbiota, metabolomic profiles, and systemic antioxidant or immune indices in non-ruminant models [15,16]. These characteristics suggest that LJP may have the potential to mitigate nutritional and oxidative challenges associated with feeding aged corn. However, to our knowledge, no study has systematically evaluated the effects of LJP supplementation on growth performance, nutrient digestibility, ruminal fermentation, rumen microbiota, and muscle antioxidant status in sheep fed aged-corn-based diets.

Therefore, the present study was conducted to evaluate the effects of dietary LJP supplementation on growth performance, nutrient digestibility, carcass traits, meat quality, ruminal fermentation characteristics, rumen microbiota, and muscle antioxidant status in Hu sheep fed aged-corn-based diets. We hypothesized that aged corn would impair nutrient utilization, ruminal fermentation activity, and antioxidant status, whereas dietary LJP supplementation would partially alleviate these adverse effects by improving ruminal fermentation and maintaining oxidative balance. This study may provide practical information on the biological responses of sheep to aged-corn-based diets and the potential application of LJP as a functional additive for improving the utilization of aged corn in sheep production.

## 2. Materials and Methods

This experiment was conducted at the Acheng Experimental Base of Northeast Agricultural University from June to August 2023. All animal procedures were reviewed and approved by the Animal Ethics Committee of Northeast Agricultural University (approval no. NEAUEC20230243). The experiment was performed in accordance with the Guidelines for Animal Research of Northeast Agricultural University, and all efforts were made to ensure animal welfare throughout the feeding trial and sample collection procedures.

### 2.1. Animals, Experimental Design, and Diets

A total of 21 healthy five-month-old male Hu sheep with similar initial body weights (39.05 ± 3.55 kg) were randomly assigned to three dietary treatments before the start of the trial, with seven sheep per treatment: (1) a normal-corn diet (NC); (2) an aged-corn diet (AC), in which normal corn was completely replaced with aged corn; and (3) the AC diet supplemented with LJP (AC+LJP). The trial lasted 70 d, consisting of a 14-d adaptation period followed by a 56-d experimental period. During the adaptation period, the concentrate-to-roughage ratio was gradually increased to 70:30 on a DM basis to allow dietary adaptation.

Throughout the experiment, each sheep was housed individually in a 1 m × 2 m pen. The roughage and concentrate were offered separately rather than as a total mixed ration. Chinese wildrye was used as the sole roughage source and accounted for 30% of dietary DM. All other dietary ingredients constituted the concentrate mixture, resulting in a concentrate-to-roughage ratio of 70:30 on a DM basis. On a concentrate DM basis, the concentrate mixture contained 46.8% corn, 20.0% corn husk, 5.0% corn germ meal, 15.0% DDGS, 8.0% soybean meal, 1.0% beet molasses, 1.5% limestone, 0.6% slow-release NH_4_Cl, 0.6% NaCl, 0.6% CaHPO_4_, 0.5% NaHSO_4_, and 0.4% compound premix.

Sheep were fed twice daily at 07:00 and 17:00, with the roughage offered first, followed by the concentrate. Feed was offered ad libitum, and the amount offered was adjusted daily to maintain approximately 5–10% refusals. Fresh drinking water was available ad libitum throughout the experiment. For the AC+LJP treatment, LJP was first premixed with a small quantity of concentrate and then thoroughly mixed with the remaining concentrate. LJP was top-dressed at 0.5% of basal-diet DM, and the daily dose was divided equally between the two feedings.

Both normal and aged corn were obtained from grain depots near Harbin, Heilongjiang Province, China. Storage conditions complied with GB/T 29890-2013, Technical Criterion for Grain and Oil-Seeds Storage [17], including ventilation, dry environment, minimum stacking distances from the floor, roof, and walls, and appropriate stacking arrangements (“Fei” or “semi-Fei” shapes). Normal corn was stored for 1 year and aged corn for 4 years. Representative samples of both corn types were analyzed for nutrient composition, starch content, and fatty acid value (Table 1). In addition, the aged corn sample was analyzed for mycotoxin concentrations prior to diet formulation (Table 2). *Laminaria japonica* polysaccharide (purity 98%, sulfate group content 28.9%) was purchased from Qingdao Bright Moon Seaweed Bio-Health Technology Group Co., Ltd. (Qingdao, China). Experimental diets were formulated to meet the nutrient requirements recommended by the National Research Council [18], and the AC+LJP diet was prepared by top-dressing LJP at 0.5% of basal-diet DM (Table 3).

### 2.2. Experimental Procedures and Sample Collection

Growth performance was evaluated throughout the experimental period. Feed offered and refusals were recorded daily before the morning feeding (07:00 h) to calculate average daily feed intake (ADFI). Individual body weights were recorded before the morning feeding on d 1 and d 70. Average daily gain (ADG), average daily feed intake (ADFI), and feed efficiency were calculated over the 70-d trial.

Diet samples were collected weekly during the experimental period and stored at −20 °C. At the end of the experiment, samples were pooled within treatment, dried at 65 °C for 48 h, ground through a 1-mm screen, and stored for chemical analysis.

Fecal samples were collected from each sheep twice daily at 07:00 and 19:00 h on d 15–16, d 40–41, and d 66–68 to obtain representative fecal samples for apparent digestibility determination. Each fecal sample was divided into two portions: one portion was preserved with 10% sulfuric acid for nitrogen fixation and subsequent nitrogen analysis, whereas the other portion was stored without preservative for the analysis of other nutrients. After all fecal collections were completed, fecal samples were composited by individual sheep, dried at 55 °C for 48 h, ground through a 1-mm sieve, and stored for determination of apparent nutrient digestibility.

At the end of the feeding trial, sheep were fasted for 16 h and transported to a commercial abattoir for slaughter. Pre-slaughter live weight was recorded immediately before slaughter. Following exsanguination and evisceration, carcass characteristics were measured, and samples of the longissimus dorsi muscle were collected between the 12th and 13th ribs. Fresh muscle samples were used for meat quality evaluation, while additional samples were stored at −20 °C for antioxidant analyses.

Immediately after slaughter, rumen contents were collected and filtered through four layers of cheesecloth. Ruminal pH was measured immediately using a calibrated portable pH meter (FiveGo F2, Mettler Toledo, Shanghai, China). Subsamples were preserved for determination of ammonia nitrogen (NH_3_-N), volatile fatty acids (VFAs), microbial crude protein (MCP), and rumen microbiota analysis. Samples for microbial analysis were snap-frozen in liquid nitrogen and stored at −80 °C until DNA extraction.

### 2.3. Laboratory Analyses

The chemical composition of the corn and experimental diets was analyzed according to standard procedures. Dry matter (DM), crude ash, crude protein (CP), and ether extract (EE) were determined following AOAC procedures [20]. Individual fecal composite samples were analyzed for DM, CP, EE, NDF, ADF, and acid-insoluble ash (AIA). Total ash was not determined for each individual fecal composite sample; therefore, fecal NFC concentrations and apparent NFC digestibility could not be calculated. Neutral detergent fiber (NDF) and acid detergent fiber (ADF) were analyzed according to the detergent fiber method described by Van Soest et al. [21], with heat-stable α-amylase and sodium sulfite used where appropriate. Non-fibrous carbohydrate (NFC) content was calculated as follows: NFC (% DM) = 100 − [CP (% DM) + EE (% DM) + ash (% DM) + NDF (% DM)]. The fatty acid value (FAV) of corn was determined according to the Chinese national standard GB/T 20570-2015 [22]. Acid-insoluble ash (AIA) was used as an internal marker to estimate the apparent digestibility of DM, CP, NDF, and ADF. Apparent nutrient digestibility was calculated using the following equation:

Apparent digestibility (%) = 100 − [(M_2n_ × M_1m_)/(M_1n_ × M_2m_)] × 100

where M_1m_ and M_2m_ represent the AIA concentrations in diet and feces, respectively, and M_1n_ and M_2n_ represent the nutrient concentrations in diet and feces, respectively.

Feed efficiency was calculated as the ratio of average daily gain to average daily feed intake and expressed as kg gain per kg feed.

Carcass evaluation included hot carcass weight, dressing percentage, back-fat thickness, eye-muscle area, and organ indices. Dressing percentage was calculated as hot carcass weight divided by pre-slaughter live weight, and organ indices were calculated as organ weight divided by pre-slaughter live weight. Meat quality traits of the longissimus dorsi muscle included pH, color coordinates (L*, a*, and b*), drip loss, cooking loss, and shear force, as commonly used indicators for evaluating lamb meat quality [23]. Muscle pH was measured at 45 min and 24 h postmortem using a calibrated portable pH meter. Meat color was determined after blooming using a CR-400 colorimeter (Konica Minolta, Tokyo, Japan), and results were expressed as CIE L*, a*, and b* values. Drip loss was determined using the suspension method described by Honikel [24]. Briefly, fresh muscle samples were weighed, suspended in sealed plastic bags without contacting the bag surface, and stored at 4 °C for 24 h. The samples were then removed, gently blotted dry, and reweighed. Drip loss was calculated as follows: drip loss (%) = [(initial weight − final weight)/initial weight] × 100.

For cooking loss determination, muscle samples were weighed, placed in heat-resistant plastic bags, and cooked in a water bath (model HH-2J, Changzhou Nuoda Instrument Technology Co., Ltd., Changzhou, China) until the internal temperature reached 70 °C. After cooling to room temperature, the samples were removed, gently blotted dry, and reweighed. Cooking loss was calculated as follows: cooking loss (%) = [(weight before cooking − weight after cooking)/weight before cooking] × 100 [24,25].

Shear force was determined using the Warner–Bratzler method. After cooking and cooling, cylindrical cores were removed parallel to the longitudinal orientation of the muscle fibers. Each core was sheared perpendicular to the fiber direction using a TA.Meat/TA.XTM meat tenderness texture analyzer equipped with a TA/WBS Warner–Bratzler standard shear blade set (Shanghai Baosheng Industrial Development Co., Ltd., Shanghai, China). The maximum force required to shear each sample was recorded in newtons [24,26].

Muscle antioxidant status was evaluated by measuring total antioxidant capacity (T-AOC), glutathione peroxidase (GSH-Px), total superoxide dismutase (T-SOD), catalase (CAT), and malondialdehyde (MDA). All indices were determined using commercial assay kits manufactured by Nanjing Jiancheng Bioengineering Institute (Nanjing, China). T-AOC was measured using a colorimetric ferrous–phenanthroline method at 520 nm (catalog no. A015-1); GSH-Px activity was determined using a DTNB colorimetric method at 412 nm (catalog no. A005-1); T-SOD activity was determined using the WST-1 method at 450 nm (catalog no. A001-3); CAT activity was determined using the ammonium molybdate method at 405 nm (catalog no. A007-1); and MDA concentration was determined using the thiobarbituric acid method at 532 nm (catalog no. A003-1). Absorbance was measured using a Synergy H1 microplate reader (BioTek Instruments, Winooski, VT, USA). All assays were performed according to the manufacturer’s instructions and the procedures described by Zhang et al. [27].

Ruminal ammonia nitrogen (NH_3_-N) concentration was determined using the indophenol blue colorimetric method. Volatile fatty acid (VFA) concentrations, including acetate, propionate, isobutyrate, butyrate, isovalerate, and valerate, were determined by gas chromatography using a GC-2010 system (Shimadzu, Kyoto, Japan) equipped with a flame ionization detector (FID), which is a widely used analytical approach for ruminal VFA quantification [28]. Briefly, rumen fluid samples were centrifuged using a refrigerated centrifuge (model Multifuge X1R, Thermo Fisher Scientific, Waltham, MA, USA), and the supernatant was filtered through a 0.22-μm membrane before analysis. Individual VFAs were separated using a capillary gas chromatography column (model HP-INNOWAX 19091N-133, Agilent Technologies, Santa Clara, CA, USA) under programmed oven temperature conditions and quantified using external standards. Total VFA concentration was calculated as the sum of individual VFA concentrations. Microbial crude protein (MCP) concentration was determined using a colorimetric method according to previously described procedures.

Microbial genomic DNA was extracted from rumen fluid samples using a commercial DNA extraction kit (D1700, Beijing Solarbio Science & Technology Co., Ltd., Beijing, China) according to the manufacturer’s instructions. The V3–V4 region of the bacterial 16S rRNA gene was amplified using the primer pair 341F/805R, which is commonly used for bacterial community profiling [29]. Amplicon sequencing was performed on an Illumina NovaSeq 6000 platform (Illumina Inc., San Diego, CA, USA). Raw sequencing reads were subjected to quality control, denoising, chimera removal, and amplicon sequence variant (ASV) generation using dada2 R package (v1.34.0), which enables high-resolution inference of amplicon sequence variants from Illumina sequencing data [30]. Microbiome data processing and diversity analyses were performed using QIIME 2 (v2024.5.0) [31]. Taxonomic assignment was conducted against the SILVA 138 rRNA gene database. Alpha diversity, beta diversity, and bacterial community composition were subsequently analyzed.

### 2.4. Statistical Analysis

All raw data were checked and organized using Microsoft Excel 2016 (Microsoft Corporation, Redmond, WA, USA) and analyzed using SAS software (v9.4; SAS Institute Inc., Cary, NC, USA). The individual sheep was considered the experimental unit. Data are presented as means with the standard error of the mean (SEM), unless otherwise stated. Normality was assessed using the Shapiro–Wilk test, and homogeneity of variance was evaluated using Levene’s test. Variables satisfying the assumptions of normality and homogeneity of variance were analyzed using one-way analysis of variance (ANOVA), with dietary treatment as the fixed effect. When a significant treatment effect was detected, multiple comparisons among treatments were performed using Tukey’s post hoc test. Differences were considered significant at *p <* 0.05, whereas 0.05 ≤ *p* < 0.10 was considered a tendency.

For rumen microbiota data, alpha-diversity indices were compared among treatments using one-way ANOVA or the Kruskal–Wallis test according to data distribution. Beta-diversity patterns were visualized using NMDS based on Bray–Curtis dissimilarity and interpreted descriptively. For selected bacterial genera, differences among treatments were analyzed using the Kruskal–Wallis test, followed by pairwise comparisons when appropriate. *p*-values were adjusted for multiple comparisons using the false discovery rate method.

## 3. Results

### 3.1. Production Performance

The effects of aged corn and LJP supplementation on the growth performance of Hu sheep are presented in Table 4. Compared with the NC diet, the AC diet significantly decreased ADG and G/F and increased F/G. LJP supplementation restored these indices to levels comparable with those of the NC group. Initial body weight, final body weight, and ADFI did not differ among treatments.

### 3.2. Apparent Digestibility

The effects of aged corn and LJP supplementation on apparent nutrient digestibility are presented in Table 5. Compared with the NC group, the AC group had significantly lower apparent digestibility of CP, NDF, and ADF, whereas LJP supplementation restored CP and NDF digestibility to levels comparable with those of the NC group. ADF digestibility in the AC+LJP group was intermediate between the NC and AC groups. DM digestibility tended to differ among treatments, whereas EE digestibility was unaffected.

### 3.3. Slaughter Performance

As shown in Table 6, the effects of replacing normal corn with aged corn and adding LJP on the slaughter performance of Hu sheep are presented. None of the slaughter performance indices, including live weight, carcass weight, dressing performance, back-fat thickness, and eye muscle area, were significantly affected by the dietary treatments (all *p* > 0.05).

### 3.4. Key Indicators of Meat Quality

The physicochemical properties of the longissimus dorsi muscle are presented in Table 7. Dietary treatment significantly affected pH at 24 h postmortem and tended to affect yellowness (b*), whereas the other meat quality traits were unaffected.

### 3.5. Muscle Antioxidant Indicators

The effects of replacing normal corn with aged corn and adding *Laminaria japonica* polysaccharide on antioxidant indexes in Hu sheep muscle are shown in Table 8. The GSH-Px activity in the AC group was significantly lower than that in the NC and AC+LJP groups (*p* = 0.002); the T-AOC level in the AC+LJP group was significantly higher than that in the NC and AC groups (*p* = 0.016); the MDA content in the AC group was significantly higher than that in the AC+LJP group (*p* = 0.023). There were no significant differences in T-SOD and CAT activities among the three groups (*p* > 0.05).

### 3.6. Rumen Microbiota

#### 3.6.1. Alpha and Beta Diversity of the Rumen Microbiota

**Figure 1:**
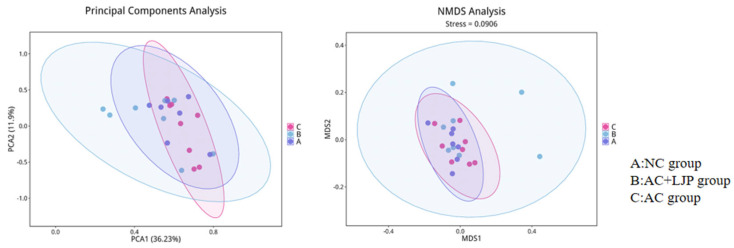
β diversity of rumen microflora in Hu sheep with aged corn replacing normal corn and adding *Laminaria japonica* polysaccharide. Note: NC group = normal corn group: 0% aged corn instead of normal corn; AC group = aged corn group: aged corn 100%, replacing normal corn; AC+LJP group = aged corn + *Laminaria japonica polysaccharide* group: aged corn 100% replacing normal corn + 0.5% *Laminaria japonica polysaccharide* antioxidant. In both panels, purple, blue, and pink symbols represent the NC, AC+LJP, and AC groups, respectively. Each point represents an individual rumen sample, and the ellipse of the corresponding color represents the distribution of samples within each treatment group.

A total of 1,487,545 high-quality 16S rRNA gene sequences were obtained, yielding 12,592 ASVs across all rumen samples. The rarefaction curves approached a plateau, indicating that the sequencing depth was sufficient to characterize the rumen bacterial communities. Dietary treatment did not significantly affect Observed_species, ACE, Chao1, or Good’s coverage (*p >* 0.05; Table 9). However, Shannon and Simpson indices tended to decrease in the AC+LJP group compared with the NC and AC groups (*p =* 0.088 and *p =* 0.092, respectively). Beta-diversity analysis based on Bray–Curtis dissimilarity showed no clear separation among the three treatments in the NMDS plot (Figure 1), suggesting that the overall rumen bacterial community structure was relatively stable across dietary treatments.

#### 3.6.2. Relative Abundance of Dominant Rumen Bacterial Genera

The relative abundance of dominant rumen bacterial genera in Hu sheep fed different diets is shown in Table 10. Among the dominant genera listed in Table 10, Lachnospiraceae_ND3007_group was significantly affected by dietary treatment (*p =* 0.0385), with a higher relative abundance in the AC group than in the AC+LJP group. Christensenellaceae_R-7_group showed a tendency to differ among treatments (*p =* 0.0634), with a numerically higher relative abundance in the AC group. Other dominant genera listed in Table 10 were not significantly affected by dietary treatment.

### 3.7. Rumen Fermentation Parameters

The rumen fermentation parameters are presented in Table 11. Dietary treatment significantly affected ruminal NH_3_-N, MCP, total VFA, acetate, isobutyrate, and butyrate concentrations (*p* < 0.05). Compared with the NC and AC+LJP groups, the AC group had lower NH_3_-N, MCP, total VFA, acetate, and butyrate concentrations. The AC+LJP group had a higher isobutyrate concentration than the other two groups. Propionate and valerate concentrations tended to differ among treatments, whereas ruminal pH, isovalerate concentration, and the acetate-to-propionate ratio were unaffected.

## 4. Discussion

In the present study, dietary aged corn reduced ADG and feed utilization efficiency without affecting ADFI, as indicated by an increased F/G and a decreased G/F. These findings suggest that the growth depression induced by aged corn was primarily associated with impaired utilization of ingested feed rather than reduced voluntary feed intake. This interpretation is further supported by the decreased apparent digestibility of CP, NDF, and ADF, together with the reduced ruminal fermentation activity observed in the AC group. Therefore, aged corn appeared to compromise growth performance mainly by limiting nutrient availability and ruminal fermentative efficiency. This interpretation is supported by recent evidence showing that stored or aged corn may undergo nutritional deterioration, including increased acidity, lipase activity, fermented and moldy grains, and reduced soluble protein during storage [32], and that dietary aged corn can alter ruminal bacterial communities and starch digestibility in dairy cows [6]. The apparent digestibility of crude protein, neutral detergent fiber, and acid detergent fiber decreased under the aged-corn diet, with a tendency toward lower dry matter digestibility. These observations suggest that reductions in fermentable carbohydrate availability and fiber degradability constrained microbial fermentation and energy extraction from the diet. In ruminants, corn starch availability and processing characteristics markedly influence ruminal fermentability, nutrient digestibility, and energy supply [1], while the dietary energy level is closely associated with nutrient digestibility, ruminal total volatile fatty acid production, and growth performance in sheep [33]. Moreover, ruminal fiber degradation has been shown to be strongly associated with feed conversion efficiency in Hu sheep, with higher NDF and ADF degradation rates contributing to improved feed utilization [34]. Consistently, sheep with higher feed efficiency exhibit greater apparent digestibility of dry matter, crude protein, NDF, and ADF than low-efficiency animals [35]. In addition, dietary rumen-degradable starch has been reported to regulate rumen fermentation, bacterial nitrogen capture, and growth performance in sheep, indicating that the balance between fermentable energy and ruminal microbial activity is critical for efficient nutrient utilization [36]. Therefore, the reduced growth performance observed in the AC group was mainly associated with impaired feed utilization and lower nutrient digestibility, together with reduced ruminal fermentation activity.

The physiological consequences of feeding aged corn appear to reflect both nutritional limitations and oxidative deterioration. Although the calculated dietary NFC concentrations were similar among treatments, the aged corn contained less starch than the normal corn. Therefore, the reductions in nutrient digestibility and ruminal VFA production in the AC group may have been related to changes in starch availability, fermentability, or physicochemical properties caused by prolonged storage, rather than to a marked difference in total dietary NFC concentration [1,6]. Starch is a major fermentable carbohydrate in corn and provides the substrate for ruminal microbial growth and VFA production [1,36,37,38]. However, starch digestibility was not directly measured, and the original analytical protocol did not include all individual-animal fecal measurements required to calculate apparent NFC digestibility. Therefore, the present study could not determine whether the observed responses were attributable to reduced starch digestibility, altered ruminal degradation kinetics, or other storage-related changes in carbohydrate availability. Accordingly, the possible involvement of altered NFC or starch utilization should be interpreted as a biologically plausible explanation rather than a directly demonstrated mechanism. Mycotoxins were analyzed only in the aged-corn sample, precluding a direct comparison between the two corn sources. Although all measured concentrations in aged corn were below the regulatory limits, possible contributions from unmeasured between-source differences in mycotoxin exposure cannot be completely excluded. Concurrently, the higher fatty acid value of aged corn indicates storage-related lipid hydrolysis and potential oxidation, which may increase reactive oxygen species production and impose oxidative stress on the gastrointestinal tract [39,40]. Although lipid oxidation products and protein oxidation were not directly measured in this study, the lower starch content and higher fatty acid value of aged corn, together with the reductions in nutrient digestibility and ruminal fermentation, may reflect combined nutritional limitation and potential oxidative deterioration. Recent reviews have highlighted that feed quality, including starch availability and oxidative stability, critically affects rumen microbial efficiency and host metabolic performance [7,41,42]. Consequently, the performance decrements observed under aged-corn feeding likely arose from an integration of nutritional deficits and oxidative stress.

LJP supplementation improved feed utilization efficiency, as evidenced by restored ADG, reduced F/G, and increased G/F, without affecting ADFI. This response indicates that LJP enhanced the efficiency with which ingested feed was converted into body weight gain, rather than stimulating feed intake. The improvement in feed utilization efficiency was likely associated with enhanced nutrient digestion and more active ruminal fermentation, as reflected by the increased apparent digestibility of CP and NDF and the elevated concentrations of NH_3_-N, MCP, TVFA, acetate, and butyrate in the AC+LJP group. Similar growth- and digestion-promoting effects of LJP have been reported in animal feeding studies, including improved ADG and digestive enzyme activity in weaned piglets [16]. Concomitantly, key ruminal fermentation markers, including ammonia nitrogen, microbial crude protein, total volatile fatty acids, acetate, and butyrate, were significantly elevated in the LJP group relative to the aged-corn group. Volatile fatty acids, particularly acetate and butyrate, are not only major energy precursors for host metabolism but also indicators of cellulolytic and saccharolytic microbial activity [7,43,44]. These results are consistent with recent studies showing that marine polysaccharides can act as fermentable or microbiota-modulating substrates and selectively influence microbial populations involved in carbohydrate fermentation, thereby promoting short-chain fatty acid production and microbial metabolic activity [14,45]. Moreover, studies in ruminants have shown that balancing rumen-degradable carbohydrate and protein supply can improve rumen nitrogen utilization and microbial protein synthesis, supporting the interpretation that improved fermentation efficiency contributed to the performance response [46]. Therefore, the improved growth efficiency observed in the AC+LJP group was associated with greater nutrient digestibility and more active ruminal fermentation. However, the specific microbial taxa and metabolic pathways underlying these responses require further verification using metagenomic, transcriptomic, or metabolomic approaches.

The rumen microbiota results provide additional insight into the fermentation responses, although they should be interpreted cautiously. The absence of significant differences in richness-related alpha-diversity indices and the lack of clear separation in beta diversity indicate that neither aged corn nor LJP markedly altered the overall rumen bacterial community structure. The decreasing tendencies in the Shannon and Simpson indices in the AC+LJP group may reflect potential alterations in community evenness or the distribution of dominant taxa, rather than evidence of a general decline in bacterial richness. Thus, the improvements in nutrient digestibility and VFA production may have resulted from changes in the activity or relative contribution of specific microbial populations without extensive restructuring of the overall community. Among the selected genera, the relative abundance of Lachnospiraceae_ND3007_group was lower in the AC+LJP group than in the AC group, whereas Christensenellaceae_R-7_group showed only a tendency to differ among treatments. Some members of the Lachnospiraceae and Christensenellaceae families have been associated with carbohydrate metabolism, fiber digestion, and ruminal VFA profiles in ruminants [47,48]. However, the direction and magnitude of these associations may vary with diet composition, host species, and experimental conditions [49]. Therefore, the observed taxonomic shifts should be considered diet-associated microbial responses rather than direct evidence that these genera mediated the beneficial effects of LJP. Because 16S rRNA amplicon sequencing primarily describes taxonomic composition and does not directly measure microbial metabolic activity, functional metagenomic analyses and targeted measurements of microbial activity are needed to clarify the contribution of these taxa to ruminal fermentation [50].

The increase in branched-chain volatile fatty acids, such as isobutyrate and valerate, in the AC+LJP group suggests enhanced amino acid catabolism and the provision of growth factors for fiber-degrading microbes, aligning with improved NDF digestibility [51,52]. Muscle antioxidant status was also improved in the AC+LJP group, as indicated by higher total antioxidant capacity, lower malondialdehyde concentration, and partial recovery of GSH-Px activity. These changes are consistent with improved muscle antioxidant status under aged-corn feeding conditions. Previous studies have reported that seaweed-derived polysaccharides and brown-algae polysaccharides can improve redox balance and antioxidant enzyme-related responses in livestock and animal models [53]. However, signaling pathways related to antioxidant regulation, such as Nrf2/Keap1 or Nrf2/ARE, were not measured in the present study. Therefore, the antioxidant-related effects of LJP should be interpreted based on the observed muscle antioxidant indices rather than as direct evidence of specific molecular signaling activation.

Despite improvements in metabolic and oxidative parameters, LJP supplementation did not significantly alter carcass weight, dressing percentage, back-fat thickness, eye muscle area, or conventional meat quality attributes, including L*, a*, drip loss, cooking loss, and shear force, which is consistent with recent lamb studies showing that phytogenic or functional feed additives may improve antioxidant status, nutrient digestibility, or energy-related metabolism without uniformly changing carcass traits or physicochemical meat-quality indices [54,55,56]. The AC+LJP group showed a higher pH at 24 h postmortem than the NC and AC groups. However, this isolated change did not translate into consistent improvements in water-holding capacity, tenderness, or meat color. Because postmortem pH is influenced by multiple factors and can interact with other meat-quality traits in a complex manner, the biological relevance of this isolated pH change requires further study. These results highlight that short-term enhancements in nutrient digestibility, ruminal fermentation, and muscle antioxidant status may not suffice to induce macroscale changes in carcass yield or meat quality; meta-analytic and experimental evidence indicates that responses to plant bioactives vary with dose, compound type, basal diet, physiological stage, and feeding duration [57,58]. Longer feeding periods, larger sample sizes, and additional measures of meat quality, such as lipid oxidation kinetics, proteomic profiling, and shelf-life assessments, are required to determine whether LJP-induced improvements in oxidative status can eventually manifest in consumer-relevant traits, especially given that *Laminaria japonica* and its polysaccharide fractions have documented antioxidant and immunomodulatory activities but limited direct evidence linking them to improved carcass or meat-quality outcomes in finishing ruminants [45,59]. Thus, under the present experimental conditions, the benefits of LJP supplementation are primarily metabolic and fermentative, rather than directly enhancing carcass or meat quality.

Several limitations of the present study should be acknowledged. First, the relatively small sample size and the 70-d trial conducted at a single experimental site may limit the generalizability of the findings. Second, only one LJP supplementation level was evaluated, and a normal-corn diet supplemented with LJP was not included; therefore, the dose–response relationship and whether the effects of LJP were specific to aged-corn-based diets could not be determined. Third, starch digestibility was not measured, and apparent NFC digestibility could not be calculated because total ash was not determined for each individual fecal composite sample. In addition, lipid and protein oxidation products were not directly measured, 16S rRNA amplicon sequencing did not assess microbial functions or metabolic activity, and mycotoxins were measured only in the aged corn. Finally, the corn sources represented only one geographical origin and one storage duration. Future studies incorporating larger sample sizes, longer feeding periods, multiple LJP doses, additional corn sources, direct carbohydrate-digestibility measurements, and functional multi-omics analyses are needed to validate and extend these findings.

## 5. Conclusions

Under the conditions of the present study, replacing normal corn with aged corn impaired growth efficiency, nutrient digestibility, ruminal fermentation, and muscle antioxidant status in Hu sheep, while carcass traits and most conventional meat quality traits were largely unaffected. Dietary supplementation with 0.5% LJP partially alleviated these adverse responses, as indicated by improved ADG, G/F, CP and NDF digestibility, ruminal NH_3_-N, MCP, TVFA, acetate, butyrate, and muscle antioxidant indices. These findings suggest that LJP may be a promising functional additive for improving the utilization of aged-corn-based diets in sheep. Further studies using dose–response designs, a normal-corn plus LJP control, and direct validation of ruminal microbial and metabolic mechanisms are warranted.

## Figures and Tables

**Table 1:** Nutrient composition and fatty acid value of normal and aged corn.

Item	Normal Corn	Aged Corn
Moisture (%)	13.42	12.70
Ash (%)	1.43	1.51
Ether extract (%)	3.82	3.50
Crude protein (%)	7.76	7.68
Neutral detergent fiber (%)	13.75	12.99
Acid detergent fiber (%)	2.92	2.45
Starch (%)	84.20	78.12
Fatty acid value (mg KOH/100 g)	53.30	84.15

Note: Nutrient levels are analyzed values.

**Table 2:** Mycotoxin concentrations measured in the aged corn used in the experiment and regulatory limits specified in GB 13078-2017 [19].

Item	Measured Value in Aged Corn (AC)	Regulatory Limit (GB 13078-2017)
Aflatoxin B1	3.6 μg/kg	≤30 μg/kg
Zearalenone (ZEN)	0.12 mg/kg	≤0.5 mg/kg
Deoxynivalenol (DON)	0.62 mg/kg	≤5 mg/kg
Ochratoxin A	<2.0 μg/kg	≤100 μg/kg
T-2 toxin	0.15 mg/kg	≤0.5 mg/kg
Fumonisins	0.85 mg/kg	≤60 mg/kg

Note: Mycotoxin concentrations were measured only in the aged corn sample. All measured values in the aged corn were below the maximum limits specified in GB 13078-2017.

**Table 3:** Ingredient and nutrient composition of the experimental diets (DM basis).

Item	NC	AC	AC+LJP
Ingredient composition (% DM)			
Chinese wildrye	30	30	30
Normal corn	32.76	0	0
Aged corn	0	32.76	32.76
Corn husk	14	14	14
Corn germ meal	3.5	3.5	3.5
DDGS	10.5	10.5	10.5
Soybean meal	5.6	5.6	5.6
Beet molasses	0.7	0.7	0.7
Limestone	1.05	1.05	1.05
slow-release NH_4_Cl	0.42	0.42	0.42
NaCl	0.42	0.42	0.42
CaHPO_4_	0.42	0.42	0.42
NaHSO_4_	0.35	0.35	0.35
Compound premix	0.28	0.28	0.28
Total	100	100	100
Nutrient levels (% DM)			
DM	95.10	94.73	94.73
CP	16.22	16.78	16.78
EE	4.33	3.86	3.86
Ash	4.51	4.69	4.69
NDF	21.14	21.27	21.27
ADF	4.59	4.78	4.78
NFC	53.80	53.40	53.40

Note: NC = normal-corn diet; AC = aged-corn diet; AC+LJP = aged-corn diet supplemented with 0.5% *Laminaria japonica* polysaccharide; DM = dry matter; CP = crude protein; EE = ether extract; NDF = neutral detergent fiber; ADF = acid detergent fiber; DDGS = distillers dried grains with solubles; NFC = non-fibrous carbohydrates. The premix supplied the following amounts per kilogram of diet: vitamin A, 1500 IU; vitamin D, 200 IU; vitamin E, 15 IU; Fe, 75 mg; Cu, 50 mg; Mn, 40 mg; Zn, 50 mg; Se, 0.5 mg; I, 1.0 mg; and Co, 0.5 mg. DM, CP, EE, ash, NDF, and ADF were analyzed values. NFC was calculated as follows: NFC (% DM) = 100 − [CP (% DM) + EE (% DM) + ash (% DM) + NDF (% DM)]. NFC values represent calculated dietary concentrations and should not be interpreted as measurements of NFC availability or digestibility.

**Table 4:** Effects of aged corn and LJP supplementation on growth performance of Hu sheep.

Item	NC	AC	AC+LJP	SEM	*p*-Value
Initial body weight (kg)	39.00	39.05	39.73	2.361	0.972
Final body weight (kg)	50.36	47.23	50.19	2.182	0.539
ADG (kg/d)	0.165 ^a^	0.126 ^b^	0.153 ^a^	0.008	0.018
ADFI (kg/d)	1.45	1.47	1.41	0.027	0.919
F/G (kg/kg)	8.79 ^b^	11.67 ^a^	9.22 ^b^	0.625	0.014
G/F (kg/kg)	0.114 ^a^	0.086 ^b^	0.109 ^a^	0.007	0.019

Note: NC = normal-corn diet; AC = aged-corn diet; AC+LJP = aged-corn diet supplemented with 0.5% *Laminaria japonica* polysaccharide; LJP = *Laminaria japonica* polysaccharide; ADG = average daily gain; ADFI = average daily feed intake; F/G = feed-to-gain ratio; G/F = gain-to-feed ratio; SEM = standard error of the mean. Values are means; *n* = 7 per treatment. Means within a row with different superscript letters differ significantly (*p* ≤ 0.05).

**Table 5:** Effects of aged corn and LJP supplementation on apparent nutrient digestibility of Hu sheep.

Item (Digestibility, %)	NC	AC	AC+LJP	SEM	*p*-Value
DM	40.66	31.28	38.47	2.234	0.056
CP	45.39 ^a^	30.75 ^b^	42.37 ^a^	3.183	0.038
NDF	44.00 ^a^	34.78 ^b^	42.26 ^a^	2.015	0.038
ADF	50.44 ^a^	41.18 ^b^	45.58 ^ab^	1.759	0.028
EE	88.75	88.54	88.82	0.173	0.706

Note: NC = normal-corn diet; AC = aged-corn diet; AC+LJP = aged-corn diet supplemented with 0.5% *Laminaria japonica* polysaccharide; LJP = *Laminaria japonica* polysaccharide; DM = dry matter; CP = crude protein; NDF = neutral detergent fiber; ADF = acid detergent fiber; EE = ether extract; SEM = standard error of the mean. Values are means; *n* = 7 per treatment. Means within a row with different superscript letters differ significantly (*p* ≤ 0.05). Starch digestibility was not measured. Apparent NFC digestibility could not be calculated because total ash was not determined for each individual fecal composite sample.

**Table 6:** Effects of aged corn and LJP supplementation on slaughter performance of Hu sheep.

Item	NC	AC	AC+LJP	SEM	*p*-Value
Live weight (kg)	48.99	45.60	48.30	2.294	0.552
Carcass weight (kg)	24.15	21.98	23.15	1.587	0.638
Dressing performance (%)	49.15	48.27	47.71	1.955	0.876
Thickness of back-fat (cm)	1.25	1.13	1.26	0.073	0.323
Eye muscle area (cm^2^)	27.60	27.90	24.78	1.719	0.375

Note: NC = normal-corn diet; AC = aged-corn diet; AC+LJP = aged-corn diet supplemented with 0.5% *Laminaria japonica* polysaccharide; LJP = *Laminaria japonica* polysaccharide; SEM = standard error of the mean. Values are means; *n* = 7 per treatment.

**Table 7:** Effects of aged corn and LJP supplementation on meat quality traits of Hu sheep.

Item	NC	AC	AC+LJP	SEM	*p*-Value
L*	29.02	26.32	26.22	1.155	0.189
a*	10.11	9.71	10.18	0.347	0.577
b*	4.76	3.96	4.06	0.231	0.054
Drip loss (%)	3.63	3.47	3.45	0.565	0.973
Cooking loss (%)	28.08	30.31	28.22	2.356	0.754
pH 45 min	5.74	5.83	5.73	0.042	0.202
pH 24 h	5.48 ^b^	5.46 ^b^	5.61 ^a^	0.036	0.018
Shear force (N)	57.78	48.32	62.28	7.447	0.402

Note: NC = normal-corn diet; AC = aged-corn diet; AC+LJP = aged-corn diet supplemented with 0.5% *Laminaria japonica* polysaccharide; LJP = *Laminaria japonica* polysaccharide; L* = lightness; a* = redness; b* = yellowness; SEM = standard error of the mean. Values are means; *n* = 7 per treatment. Means within a row with different superscript letters differ significantly (*p* ≤ 0.05).

**Table 8:** Effects of aged corn and LJP supplementation on muscle antioxidant indicators of Hu sheep.

Item	NC	AC	AC+LJP	SEM	*p*-Value
T-SOD (U/mL)	10.05	9.19	9.46	0.668	0.658
CAT (U/mL)	2.23	2.04	2.36	0.142	0.303
MDA (nmol/mL)	0.36 ^ab^	0.43 ^a^	0.27 ^b^	0.036	0.023
GSH-Px (μmol/L)	9.83 ^a^	8.15 ^b^	9.27 ^a^	0.279	0.002
T-AOC (U/mL)	1.56 ^b^	1.41 ^b^	1.87 ^a^	0.104	0.016

Note: NC = normal-corn diet; AC = aged-corn diet; AC+LJP = aged-corn diet supplemented with 0.5% *Laminaria japonica* polysaccharide; LJP = *Laminaria japonica* polysaccharide; T-SOD = total superoxide dismutase; CAT = catalase; MDA = malondialdehyde; GSH-Px = glutathione peroxidase; T-AOC = total antioxidant capacity; SEM = standard error of the mean. Values are means; *n* = 7 per treatment. Means within a row with different superscript letters differ significantly (*p* ≤ 0.05).

**Table 9:** Effects of aged corn and LJP supplementation on rumen bacterial alpha diversity of Hu sheep.

Item	NC	AC	AC+LJP	SEM	*p*-Value
Observed_species	1654.57	1694.63	1425.50	127.947	0.287
ACE	1761.81	1818.20	1509.03	152.000	0.315
Chao1	1743.47	1802.73	1496.59	150.023	0.318
Shannon	9.09	9.15	8.63	0.175	0.088
Simpson	0.995	0.994	0.992	0.001	0.092
Good’s coverage	0.994	0.993	0.995	0.001	0.559

Note: NC = normal-corn diet; AC = aged-corn diet; AC+LJP = aged-corn diet supplemented with 0.5% *Laminaria japonica* polysaccharide; LJP = *Laminaria japonica* polysaccharide; ACE = abundance-based coverage estimator; Chao1 = Chao1 richness estimator; Good’s coverage = sequencing coverage; SEM = standard error of the mean. Values are means; *n* = 7 per treatment.

**Table 10:** Relative abundance of dominant rumen bacterial genera in Hu sheep fed different diets.

Item	NC	AC	AC+LJP	SEM	*p*-Value
*Prevotella*	13.17	9.38	16.14	2.49	0.1836
Rikenellaceae_RC9_gut_group	10.88	10.34	8.10	1.62	0.4663
*Succiniclasticum*	4.97	4.31	4.79	0.91	0.8764
*Selenomonas*	2.64	3.99	6.05	1.38	0.2552
Christensenellaceae_R-7_group	2.48	3.54	1.57	0.55	0.0634
Veillonellaceae_UCG-001	2.83	2.45	1.97	0.43	0.3986
Prevotellaceae_UCG-001	2.04	2.31	2.74	0.51	0.6429
NK4A214_group	2.47	2.72	1.72	0.43	0.2516
Prevotellaceae_UCG-003	1.11	1.27	1.59	0.30	0.5598
Lachnospiraceae_NK3A20_group	0.85	0.82	1.21	0.33	0.6544
*Saccharofermentans*	0.89	1.13	0.71	0.16	0.2192
*Ruminococcus*	0.66	0.93	1.07	0.16	0.2369
Lachnospiraceae_ND3007_group	0.88 ^ab^	1.17 ^a^	0.42 ^b^	0.19	0.0385
UCG-005	0.96	0.51	0.70	0.19	0.2961
*Papillibacter*	0.62	0.68	0.54	0.16	0.8438
*Desulfovibrio*	0.68	0.63	0.45	0.12	0.3971
*Butyrivibrio*	0.55	0.64	0.47	0.08	0.3269
Prevotella_7	0.11	0.41	1.07	0.41	0.2759
*Anaerovibrio*	0.52	0.59	0.50	0.18	0.9279
*Pseudobutyrivibrio*	0.57	0.49	0.42	0.07	0.4034
[Eubacterium]_ruminantium_group	0.23	0.39	0.76	0.26	0.3739
*Anaerovorax*	0.44	0.54	0.42	0.07	0.4034
UCG-004	0.30	0.57	0.49	0.13	0.3855
*Fretibacterium*	0.31	0.73	0.26	0.18	0.1584
Lachnospiraceae_XPB1014_group	0.56	0.51	0.20	0.14	0.1903
*Sharpea*	0.54	0.16	0.55	0.28	0.5348
*Quinella*	0.32	0.51	0.40	0.16	0.7256
*Treponema*	0.36	0.46	0.37	0.07	0.55
*Fibrobacter*	0.37	0.38	0.44	0.10	0.8546
Others	46.68	47.46	42.82	3.08	0.5360

Note: NC = normal-corn diet; AC = aged-corn diet; AC+LJP = aged-corn diet supplemented with 0.5% *Laminaria japonica* polysaccharide; LJP = *Laminaria japonica* polysaccharide; SEM = standard error of the mean. Values are means; *n* = 7 per treatment. Means within a row with different superscript letters differ significantly (*p* ≤ 0.05).

**Table 11:** Effects of aged corn and LJP supplementation on rumen fermentation parameters of Hu sheep.

Item	NC	AC	AC+LJP	SEM	*p*-Value
pH	6.68	6.58	6.65	0.076	0.644
NH_3_-N (mg/dL)	26.26 ^a^	15.31 ^b^	29.24 ^a^	2.727	0.013
MCP (mg/100 mL)	27.46 ^a^	22.49 ^b^	25.11 ^a^	1.244	0.037
TVFA (mmol/L)	62.26 ^a^	47.22 ^b^	74.21 ^a^	4.449	0.004
Acetic (mmol/L)	40.45 ^a^	29.78 ^b^	46.52 ^a^	2.641	0.003
Propionic (mmol/L)	13.20	11.06	16.19	1.318	0.052
Isobutyric (mmol/L)	0.87 ^b^	0.93 ^b^	2.07 ^a^	0.137	<0.001
Butyric (mmol/L)	5.79 ^a^	3.55 ^b^	6.81 ^a^	0.579	0.006
Isovaleric (mmol/L)	1.34	1.12	1.62	0.194	0.225
Valeric (mmol/L)	0.61	0.77	1.00	0.115	0.094
Acetic: Propionic	3.11	2.86	2.88	0.192	0.602

Note: NC = normal-corn diet; AC = aged-corn diet; AC+LJP = aged-corn diet supplemented with 0.5% *Laminaria japonica* polysaccharide; LJP = *Laminaria japonica* polysaccharide; NH_3_-N = ammonia nitrogen; MCP = microbial crude protein; TVFA = total volatile fatty acids; SEM = standard error of the mean. Values are means; *n* = 7 per treatment. Means within a row with different superscript letters differ significantly (*p* ≤ 0.05).

## Data Availability

All data generated or analyzed during this study are included in this published article. Additional data related to this study are available from the corresponding author upon reasonable request.
